# Strategic Engagement of Technical Surge Capacity for Intensified Polio Eradication Initiative in Nigeria, 2012–2015

**DOI:** 10.1093/infdis/jiv494

**Published:** 2016-04-02

**Authors:** Yared G. Yehualashet, Pascal Mkanda, Alex Gasasira, Tesfaye Erbeto, Anthony Onimisi, Janet Horton, Richard Banda, Sisay G. Tegegn, Haruna Ahmed, Oluwole Afolabi, Alieu Wadda, Rui G. Vaz, Peter Nsubuga

**Affiliations:** 1 World Health Organization, Country Representative Office, Abuja, Nigeria; 2 World Health Organization, Regional Office for Africa, Brazzaville, Congo; 3 World Health Organization, Country Representative Office, Monrovia, Liberia; 4 Global Public Health Solutions Atlanta, Georgia

**Keywords:** surge capacity, polio eradication initiative, polio funded personnel, World Health Organization, technical assistance, Nigeria

## Abstract

***Background.*** Following the 65th World Health Assembly (WHA) resolution on intensification of the Global Poliomyelitis Eradication Initiative (GPEI), the Nigerian government, with support from the World Health Organization (WHO) and other partners, implemented a number of innovative strategies to curb the transmission of wild poliovirus (WPV) in the country. One of the innovations successfully implemented since mid 2012 is the WHO's engagement of surge capacity personnel.

***Methods.*** The WHO reorganized its functional structure, adopted a transparent recruitment and deployment process, provided focused technical and management training, and applied systematic accountability framework to successfully manage the surge capacity project in close collaboration with the national counterparts and partners. The deployment of the surge capacity personnel was guided by operational and technical requirement analysis.

***Results.*** Over 2200 personnel were engaged, of whom 92% were strategically deployed in 11 states classified as high risk on the basis of epidemiological risk analysis and compromised security. These additional personnel were directly engaged in efforts aimed at improving the performance of polio surveillance, vaccination campaigns, increased routine immunization outreach sessions, and strengthening partnership with key stakeholders at the operational level, including community-based organizations.

***Discussion.*** Programmatic interventions were sustained in states in which security was compromised and the risk of polio was high, partly owing to the presence of the surge capacity personnel, who are engaged from the local community. Since mid-2012, significant programmatic progress was registered in the areas of polio supplementary immunization activities, acute flaccid paralysis surveillance, and routine immunization with the support of the surge capacity personnel. As of 19 June 2015, the last case of WPV was reported on 24 July 2014. The surge infrastructure has also been instrumental in building local capacity; supporting other public health emergencies, such as the Ebola outbreak response and measles and meningitis outbreaks; and strengthening the integrated disease surveillance and response. Due to weak health systems in the country, it is vital to maintain a reasonable level of the surge capacity for successful implementation of the 2013–2018 global polio endgame strategy and beyond.

In May 2012, the 65th World Health Assembly (WHA) resolved to intensify the Global Polio Eradication Initiative (GPEI) and declared the completion of poliovirus eradication a programmatic emergency for global public health [1]. The WHA further urged member states with poliovirus transmission to declare a national public health emergency. Nigeria responded by intensifying polio eradication activities, particularly in the northern states, which resulted in a 95% reduction in the number of wild poliovirus (WPV) infections in 2010 [2]. This progress was not sustained, and the country experienced a 3-fold increase in the number of WPV infections in 2011, compared with 2010 [3, 4].

In October 2011, Nigeria announced the establishment of a Presidential Task Force on Polio Eradication to provide highest-level leadership to the national effort to guide the polio eradication initiative (PEI) back on track. The task force oversaw a number of initiatives aimed at rapidly achieving the goal of interrupting poliovirus transmission within the shortest time possible. However, an in-depth review of the performance of polio eradication activities in the highest-risk states and local government areas (LGAs) indicated that the high-level commitment at federal and state level did not always translate into improved program quality at the operational level [5]. The 23rd meeting of the Expert Review Committee (ERC) on Polio Eradication and Routine Immunization in Nigeria observed that the number and geographical extent of polio cases in the country were increasing and that, as of the end of March 2012, nearly double the number of WPV cases had been reported, compared with the same period in 2011 [6].

## The Rationale for Surge Capacity

The health system in Nigeria faces multiple challenges, including poor healthcare infrastructure, underfunding of programs, frequent strikes by health workers, and weak oversight at the lower levels [7–10]. In recognition of these shortcomings, the 2012 Nigeria Polio Eradication Emergency Plan highlighted the contribution of implementing partners to boost their technical capacity towards improved PEI performance at the field level.

Furthermore, in January 2012, Nigerian high level government officials visited India to review factors that contributed to the successes in the polio eradication efforts in that country [4]. One of the lessons learnt was the positive contribution of increased immunization personnel at the local level to intensify PEI interventions at the operational level. Consequently, the Nigerian federal government and implementing partners agreed to adapt the Indian model of technical surge capacity through engagement of additional personnel in states that were prioritized on the basis of epidemiological and operational parameters [11]. This strategy was expected to accelerate the operationalization of the high-level commitment from political, traditional, and religious leaders into improved operational outcome at the LGA, ward, and settlement levels.

The World Health Organization (WHO) team in Nigeria has a broad range of public health practitioners comprising physicians, epidemiologists, pharmacists, logisticians, laboratory experts, data managers, communication experts, program managers, and administrative support staff providing technical support for polio eradication, routine immunization (RI), and accelerated control of vaccine-preventable diseases at national and subnational levels. The WHO was uniquely positioned to implement the surge project as per the mandate from 41st WHA and its physical presence in all 36 states of the federation and the federal capital territory [12]. Prior to the introduction of the surge capacity, the WHO had 756 personnel (as of January 2012) in Nigeria, with the field offices having skeletal technical and administrative structures. The staffing strength in priority states was not sufficient to meet the increasing programmatic demands.

To this end, in March 2012, WHO-Nigeria developed a project to substantially increase its technical capacity to support planning, implementation, monitoring, and evaluation of critical PEI activities, particularly in the states, LGAs, and wards (districts) at highest risk for polio transmission, to ultimately achieve interruption of poliovirus transmission within the shortest time possible.

In this article, we document the recruitment, deployment, and management processes and how implementation of the WHO's surge capacity project contributed to the improvement of PEI and RI performance indicators.

## METHODS

We conducted a retrospective review of publications by global and local PEI partners and working groups, WHO internal documents, secondary sources, and unpublished reports to obtain data for this article.

### Resource Mobilization

In early 2012, the WHO country office, with input from key stakeholders, submitted a proposal to the Bill and Melinda Gates Foundation to support the implementation of the surge capacity project. Between 2012 and 2015, the Bill and Melinda Gates Foundation allocated $84 million to support the surge capacity project.

### Project Objectives, Roles and Responsibilities

The surge capacity project focused on 11 states at high risk for polio transmission (hereafter, “HR states”), selected on the basis of an epidemiological risk analysis. The states were Bauchi, Borno, Jigawa, Kaduna, Kano, Katsina, Kebbi, Niger, Sokoto, Yobe, and Zamfara. The project aimed to support the HR states to achieve and sustain quality supplemental immunization activities (SIAs) required to ensure poliovirus transmission is interrupted, to maintain certification standard acute flaccid paralysis (AFP) surveillance and to improve RI coverage [13]. Critical milestones and detailed activities required to meet the objectives were specified in the project framework.

### Recruitment and Selection

The recruitment process commenced after developing elaborate terms of reference, key performance indicators, and qualification requirements for each position. The WHO communicated the vacancies through internal notices and existing health networks at all levels. Under its overall responsibility, the WHO formed an interagency panel comprising representatives from National Primary Health Care Development Agency, the Nigerian Ministry of Health, and the United Nations Children's Fund to conduct the selection process. The screening included short-listing the applications on the basis of standard scoring criteria, administration of pretraining and posttraining tests, and interviews. Each panel submitted a detailed report on the selection proceedings, along with a list of recommended candidates and their scores, to the WHO central office for approval.

### Mode of Engagement and Deployment

The WHO adopted various contract types to facilitate deployment of the surge capacity personnel in the prevailing epidemiological, operational, and security circumstances. Such contract types included staff contracts, characterized by short or fixed terms and mainly involved for central and zonal level staff; special services agreements, which involved nonstaff, were governed by United Nations security regulations, and were used mainly to engage cluster coordinators; and agreements for performance of work, which involved nonstaff (ie, LGA facilitators, field volunteers, and data assistants), were not governed by UN security restrictions, and allowed flexibility in determining the payment rates and movement of the contract holder.

### Training

In addition to the training conducted to administer selection tests during the recruitment process, the WHO and partners organized and cascaded trainings. The training package covered topics on the Expanded Program on Immunization and involved field visits to health facilities offering RI and surveillance services, to further perform hands-on practices using the approved RI and surveillance checklists.

In 2012–2013, the WHO engaged an international consulting firm and conducted a series of management trainings for top- and middle-level managers, including surge capacity personnel with supervisory responsibilities. The training introduced the following concepts: understanding management styles, performance management and accountability, team building and maintenance, objective setting and monitoring performance, handling difficult conversations, coaching for development, and improving creativity and innovative problem solving skills.

### Rotation and Redeployment

The WHO officers support efforts to enhance program ownership and oversight, improve immunization operations, and sustain high quality surveillance performance. It is important to provide them with a working environment conducive to providing objective technical and operational guidance to the PEI program. The WHO operational focus is drawn from the national immunization plan, which drives the allocation and deployment of technical assistance, particularly at the field levels. To this end, after close review of the WHO's staffing strength vis-à-vis the expected technical deliverables, the WHO implemented a series of in-country staff rotation.

The WHO used the policy of placing high-quality personnel in the worst-performing LGAs. To this effect, based on a thorough risk analysis conducted in collaboration with national authorities and partners, coupled with the performance assessment results, state- and LGA-level personnel were reassigned biannually to deploy high performers in priority areas (WHO/UNICEF, polio funded personnel deployment optimisation report, 2014).

### Performance Accountability Framework

Along with the substantial increase in the number of personnel, it was necessary to strengthen the monitoring and evaluation capability to systematically monitor staff performance. In 2014, the WHO introduced a systematic accountability framework and implemented it in all its field offices, using key performance indicators aided by geographical information systems and mobile device technologies, coupled with periodic supportive supervisory visits to the field (WHO Nigeria, Quarterly performance report of accountability framework, 2014). Additional monitoring and evaluation officers, as well as data management personnel, were hired at the central level and in priority zones and states.

## RESULTS

### Adjustment on WHO Functional Structure

In March 2012, the WHO made a major adjustment to its functional organogram to establish that adequate technical, managerial, and administrative support systems at all levels to effectively support the enhanced WHO technical infrastructure. The revised structure reflected the WHO's extended presence down to LGA and ward levels after the introduction of the surge capacity. The adjustment further optimized the span of control and supervisory lines.

### Capacity Building

In 2013, the WHO trained 532 cluster coordinators and LGA facilitators and 1 637 field volunteers. Each session took 4 days, using the Expanded Program on Immunization comprehensive package that included polio SIAs, nonpolio SIAs, surveillance, RI, and data management. Specific topics were drawn from the standard operating procedures developed for technical surge personnel (WHO Nigeria, Standard Operating Procedure for immunization cluster personnel, 2013). Furthermore, 214 participants received management training, of whom 45 were from government and partner agencies.

### Engagement and Deployment

Following the full implementation of the surge capacity, the human resource strength of the WHO increased by >400%. Table 1 shows that 91% of the 2 677 personnel were deployed in the northern part of the country, which includes the 11 HR states. The remaining states with inadequate technical capacity were allocated limited additional support to enable them to maintain their polio-free status. Zonal offices and central levels were also strengthened with basic expertise to equip them with adequate oversight and program support capabilities. The majority (81%) of the personnel funded for polio-related activities were deployed to support the implementation of PEI and RI strategies at LGA and ward levels.

**Table 1.** Deployment of World Health Organization Personnel Funded for Polio Activities, Including Surge Capacity, by Zone and Contract Type, May 2015

Description	Deployment Level					Contract Type			
	Ward (FVs)	LGA (LGAFs)	State Level	Central Level	Total	Staff Contract	SSA	APW	Total
North-west zone	1 116	253	152	…	1 521	60	84	1 377	1 521
North-east zone	448	121	97	…	666	50	41	575	666
North-central zone	132	65	70	…	267	41	27	199	267
Southern zones	…	29	115	…	144	85	26	33	144
Central level	…	…	…	79	79	61	18	…	79
Total	1 696	468	434	79	2 677	297	196	2 184	2 677

Abbreviations: APW, agreement for performance of work (independent consultancy contract); FV, field volunteer; LGA, local government area; LGAFs, local government area facilitators; SSA, special service agreement (nonstaff contract).

The WHO used agreements for performance of work for >80% of the overall personnel, while 11% and 7% of personnel were engaged under fixed-term and special service agreements, respectively. These cadres possessed a higher technical caliber and were placed in supervisory responsibilities at state, zonal, and central levels.

Between August 2013 and July 2014, the WHO undertook various waves of mass rotations that resulted in rotation of 50 high-level technical officers, the majority of whom coordinated zonal and state level activities. The administrative focal points in all 37 field offices were also relocated or reprofiled between December 2014 and February 2015. These periodic movements contributed to enhanced managerial capabilities and fostered staff accountability.

**Figure 1.**
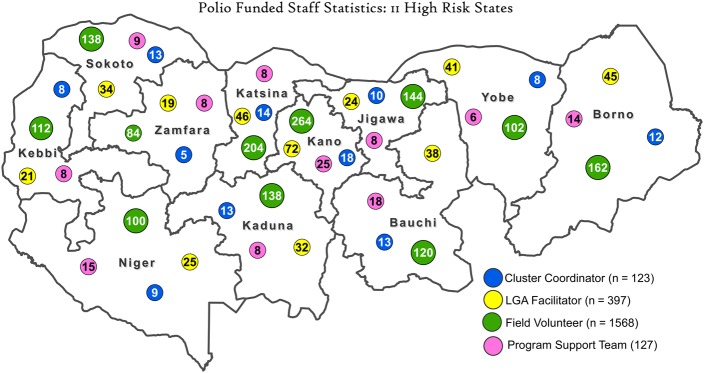
Deployment of World Health Organization personnel funded for polio activities as part of the surge capacity across Nigerian states at high risk for polio transmission, May 2015. The program support team includes those who support data, laboratory, administrative, security, and finance functions. Abbreviation: LGA, local government area.

Guided by surge capacity deployment optimization analysis and the cumulative feedback during 2014 from the accountability framework, the WHO made necessary adjustments during the 2015 surge capacity deployment. States in northeast zone with persisting security challenges and programmatic priorities were assigned more personnel by shifting staff from states such as Jigawa that had relatively more resources. The current deployment of 2 210 human resources involved in the surge capacity in the 11 HR states is shown in Figure 1. Along with this, the 23rd ERC assessed Borno, Kano, Sokoto, and Yobe states' immunization coverage during campaigns as stagnant or very slow. These states were, therefore, accorded higher attention in the allocation of surge capacity.

### Contribution Toward Improved PEI and RI Performance Indicators

The majority of surge capacity personnel were deployed and became fully functional toward the end of 2012. As highlighted in Figures 3–6, the key surveillance, polio SIA, and RI indicators that were used for the surge capacity project assessment framework showed improvement after 2012.

**Figure 2.**
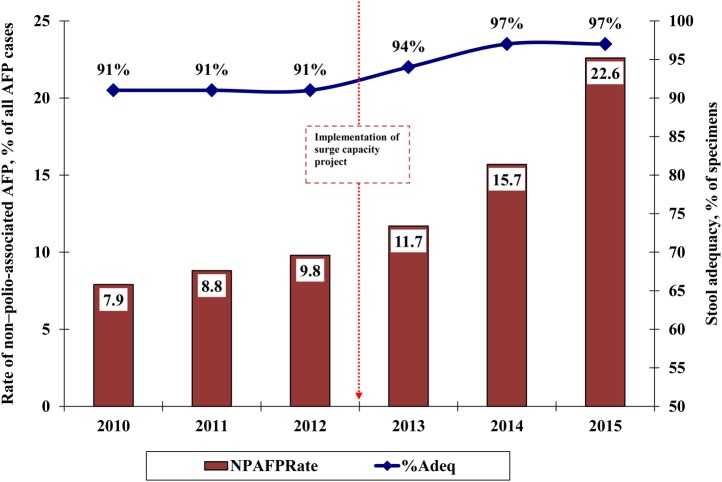
Trend of the 2 core acute flaccid paralysis (AFP) surveillance indicators—non–polio-AFP rates (red bars) and stool adequacy (blue line)—in 11 Nigerian states at high risk for polio transmission, 2010–2015. Data for 2015 were collected up to August. Stool adequacy refers to stool specimens in good condition collected from individuals with AFP ≤14 days after onset. The target threshold for stool adequacy is ≥80% of specimens. Source: World Health Organization, Nigeria Country Office, Abuja.

Figure 2 illustrates that 2 core AFP surveillance indicators—the non–polio AFP rate and stool adequacy—showed marked improvement after the introduction of the surge capacity. Although the program had achieved the threshold target of 2 non–polio-associated AFP cases per 100 000 persons aged <15 years and 80% stool adequacy, with the implementation of the surge capacity the program could report more AFP cases with a high proportion of adequate stool specimens and a higher non–polio-associated AFP rate. Furthermore, with direct involvement of the surge capacity personnel, the number of LGAs that did not meet the 2 AFP surveillance indicators significantly reduced, from 55 in 2012 to only 4 in August 2015.

**Figure 3.**
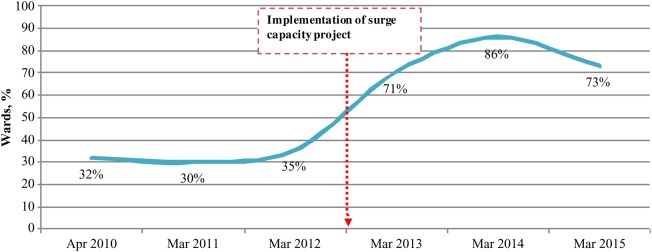
Proportion of wards with updated microplans in 11 Nigerian states at high risk for polio transmission, 2010–2015. For the purpose of this analysis, the March immunization round is selected for each year because the major microplan activities are conducted throughout the country. The slight reduction in 2015 is attributed mainly to a low level of microplanning activities in Borno state, owing to the inaccessibility of wards in a number of local government areas for security reasons. Source: World Health Organization, Nigeria Country Office, Abuja.

**Figure 4.**
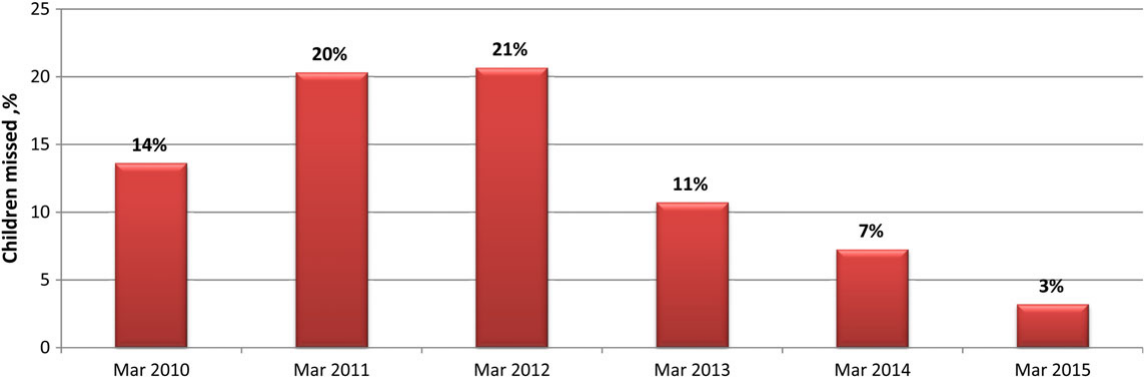
Trend of the proportion of wards in 11 Nigerian states at high risk for polio transmission where >10% of children were missed by vaccination campaigns, 2010–2015. For the purpose of this analysis, the March immunization round is selected for each year because major microplanning activities are conducted throughout the country. Source: World Health Organization, Nigeria Country Office, Abuja.

Microplan development is one of the core process indicators for successful and quality immunization campaigns for which the surge capacity personnel are directly responsible. As Figure 3 shows, the proportion of wards with an updated microplan dramatically increased from 2013 onward. As a result of an improved microplan and other innovative interventions, the proportion of children missed by vaccination campaigns decreased substantially, and the program could reach more children. The proportion of wards with >10% children missed by vaccination campaigns decreased from 21% in 2012 to 3% in 2015 (Figure 4).

As of 19 June 2015, cases of WPV infection in the country decreased during 2012–2014, with 122 cases in 2012, 53 cases in 2013, and 6 cases in 2014, with the last case reported on 24 July 2014. End-process independent monitoring of polio SIAs showed that 86% of wards achieved 90% vaccine coverage by the end of 2012, which increased to 96% by the end of 2014.

**Figure 5.**
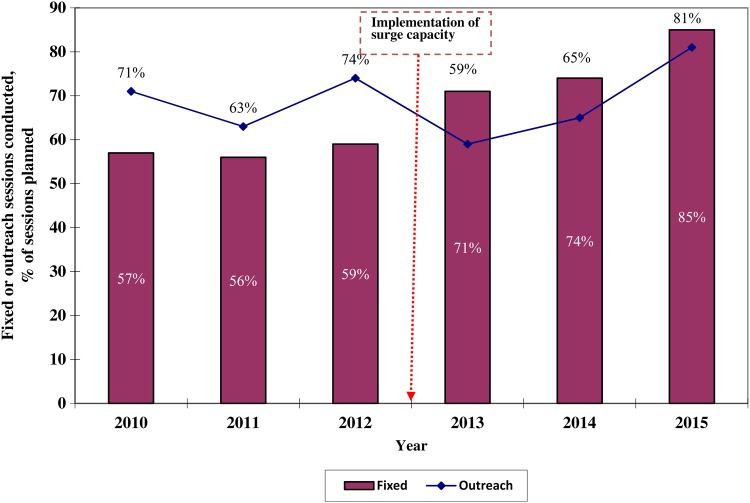
Comparison of fixed and outreach sessions conducted among the total number planned in 11 Nigerian states at high risk for polio transmission , 2010–2015. Data for 2015 were collected up to August. Source: World Health Organization, Nigeria Country Office, Abuja.

**Figure 6.**
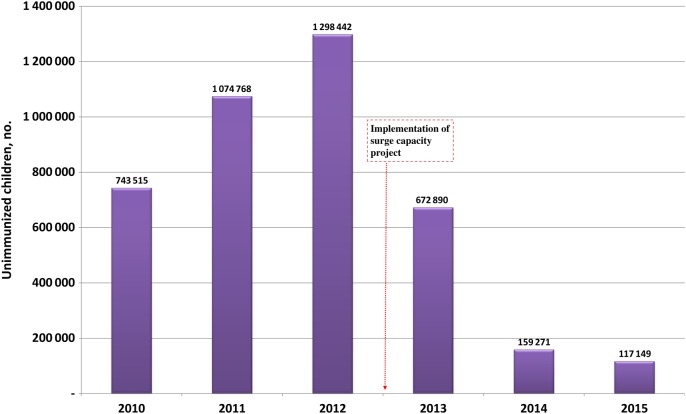
Number of unimmunized children in 11 Nigerian states at high risk for polio transmission, 2010–2015. Source: World Health Organization, Nigeria Country Office, Abuja.

With respect to RI indicators, the surge capacity also contributed to the steady improvement in the implementation of fixed and outreach sessions to immunize children. Each state has a goal of conducting 80% of planned fixed and outreach RI sessions. As Figure 5 demonstrates, the proportion of sessions conducted improved after 2012, and the target was met in 2015 (based on data collected up to August). As shown in Figure 6, in conjunction with other factors, the number of unimmunized children decreased from 1.3 million in 2012 to approximately 120 000 in 2015 in the 11 HR states.

## DISCUSSION

The WHO reorganized its functional structure, adopted a transparent recruitment and deployment process, provided focused technical and management training, and applied systematic accountability framework to successfully manage the surge capacity project in close collaboration with the national counterparts and partners. The engagement of >2 200 surge capacity personnel has enhanced the WHO's technical capability to contribute to the achievements registered in the intensified PEI and RI activities in Nigeria. Guided by the epidemiological risk analysis and feedback from the accountability framework, the majority (92%) of the surge personnel were strategically deployed in the northern part of the country, which remains a priority from epidemiological, operational, and security perspectives. To facilitate acceptance by the community, the surge capacity personnel were hired from the communities where they resided, using suitable contractual arrangement. The personnel have built up personal relationships with key stakeholders in the community to obtain buy-in for successful implementation of several innovative interventions that have been introduced since 2012.

The surge project gave the program an opportunity to use technically competent health professionals who understand the grassroots' cultural and operational context. As such, they are suited to address challenges unique for every local area in the context of the diverse political, social and economic landscape in Nigeria. Their extended presence at LGA and ward levels created the capacity to successfully translate the high-level commitments and strategies into operational levels.

The surge capacity is well aligned with the updated WHO functional structure and integrated into the existing health systems in the country. The collaboration with the government and partners in recruitment, deployment of the surge personnel, and collective program reviews at ward, LGA, state, and national levels, coupled with application of WHO's rigorous accountability framework, contributed toward improved program performance. As outlined in the WHO's standard operating procedures, the surge personnel are directly involved in training, planning, implementation, monitoring, and review of the field-level polio campaign performance and timely outbreak investigation and response; active case based surveillance; and intensification of RI activities (WHO Nigeria, Standard Operating Procedure for immunization cluster personnel, 2013). Furthermore, the surge capacity personnel have been instrumental in sustaining the program in security-compromised areas, which were not accessible to regular staff.

Owing to the weak health system in the country, the surge personnel are also called upon to support other activities, such as nonpolio immunization campaigns, integrated disease surveillance and response, and other disease outbreak investigations and responses [8].

The surge project faces challenges of attrition, mainly because of the implementation of accountability framework and, to some degree, resignations. In 2014, 300 agreements for performance of work were not renewed, because of persistent poor performance (WHO Nigeria, Quarterly performance report of accountability framework, 2014) [14]. The WHO manages staff turnover by maintaining a roster of potential replacements. The surge project caused internal brain drain to a certain extent, as the personnel were hired from the existing health system pool, particularly from local government institutions. The effect was mitigated because personnel recruited during the surge capacity were redeployed in the same area from which they were recruited and were hired to support a program similar to what they previously supported, although they were managed under WHO's systematic accountability framework, with provision of a better incentive scheme. The surge project required substantial financial commitment and was externally funded. Financial sustainability remains one of the major concerns. The WHO and partners are engaged in continued dialogue to sustain the project optimally for successful implementation of the polio endgame strategy.

Despite the challenges in recruitment and maintenance of such a large workforce, the partnership coordination and management architecture put in place contributed to maximize efficiency and accountability from ward to national levels [15].

In conclusion, as recommended in the 24th ERC report and the 2012–2013 global polio emergency plan, the WHO responded to the call for action and equipped its personnel, including those involved in the surge capacity, with the standardized tools and knowledge to optimize the substantial investment on the project [15]. As stated in the 2012–2014 Nigeria Polio Eradication Emergency Plans, GPEI independent monitoring board reports, and ERC recommendations, the engagement of the surge capacity was one of the several innovative strategies that contributed toward the recent PEI and RI achievements [16–18].

We recommend the sustenance of the surge project to maintain the polio program's momentum and successful implementation of the polio endgame strategy in Nigeria [19, 20]. As part of polio legacy planning the government and partners should consider leveraging the polio surge capacity infrastructure to support health systems strengthening in general and new vaccine introductions and vaccine-preventable disease surveillance activities in particular [20].
